# Peripheral nervous system adverse events associated with immune checkpoint inhibitors

**DOI:** 10.1007/s00415-023-11625-1

**Published:** 2023-02-17

**Authors:** Simone Rossi, Francesco Gelsomino, Rita Rinaldi, Lorenzo Muccioli, Francesca Comito, Alessandro Di Federico, Andrea De Giglio, Giuseppe Lamberti, Elisa Andrini, Veronica Mollica, Roberto D’Angelo, Flavia Baccari, Corrado Zenesini, Pierandrea Madia, Emanuel Raschi, Pietro Cortelli, Andrea Ardizzoni, Maria Guarino

**Affiliations:** 1 IRCCS Istituto delle Scienze Neurologiche di Bologna, Via Albertoni 15, 40138 Bologna, Italy; 2 Department of Biomedical and Neuromotor Sciences (DIBINEM), Alma Mater Studiorum–University of Bologna, Bologna, Italy; 3 Medical Oncology, IRCCS Azienda Ospedaliero-Universitaria di Bologna, Bologna, Italy; 4 Department of Experimental, Diagnostic and Specialty Medicine (DIMES), Alma Mater Studiorum-University of Bologna, Bologna, Italy; 5 Pharmacology Unit, Department of Medical and Surgical Sciences, Alma Mater Studiorum-University of Bologna, Bologna, Italy

**Keywords:** Neuromuscular toxicities, Myasthenia gravis, Myositis, Autoimmune neuropathies, ICI rechallenge

## Abstract

**Background:**

Immune checkpoint inhibitors (ICIs) represent an effective cancer immunotherapy yet are associated with immune-related adverse events (irAEs). The aim of this study was to characterize irAEs involving the peripheral nervous system (PNS-irAEs) in a real-world cohort of ICI-treated patients.

**Methods:**

Cancer patients treated with ICIs between January 2014 and March 2022 were included. Patients with PNS-irAEs were identified and divided into two groups: (1) cranial/peripheral neuropathies and (2) myasthenia gravis (MG) and/or myositis. Clinical characteristics and outcomes, measured with the modified Rankin Scale (mRS), were compared among the two groups.

**Results:**

Among 920 ICI-treated patients, 20 patients (2.17%) developed a PNS-irAEs. The median latency from ICI exposure was 8.8 weeks and the median time from onset to clinical nadir was 3.5 weeks. Eleven patients developed a neuropathy: polyneuropathy (*n* = 4), cranial neuropathy (*n* = 3), small-fiber neuropathy (*n* = 3), brachial plexopathy (*n* = 1). Nine patients presented MG and/or myositis: concomitant MG and myositis (*n* = 6), isolated myositis (*n* = 2), exacerbation of MG (*n* = 1). Immunosuppressive treatment and/or ICI withdrawal determined a significant clinical improvement, expressed by a mRS reduction, in the neuropathy group (*p* = 0.004), but not in the MG/myositis group (*p* = 0.11). Overall, death due to irAEs occurred in four patients (20%), all with MG/myositis. Compared to patients with neuropathies, those with MG/myositis had a shorter latency onset (*p* = 0.036), developed more frequently concomitant non-neurologic irAEs (*p* = 0.028) and showed a higher mortality rate (*p* = 0.026).

**Conclusions:**

In our large cohort of ICI-treated patients, 2.17% developed PNS-irAEs. Compared to ir-neuropathies, ir-MG/myositis tend to occur earlier from ICI exposure and present a worse response to treatment and a higher mortality.

## Introduction

The introduction of immune checkpoint inhibitors (ICIs) into the clinical practice represented a breakthrough in the management of patients with cancer, and ICIs are now regarded as a “standard of care” treatment for several types of cancer [1]. ICIs are monoclonal antibodies that bind intrinsic cell-surface molecules involved in immune system downregulation. In particular, ipilimumab targets cytotoxic T-lymphocyte antigen 4 (CTLA-4), pembrolizumab, nivolumab, and cemiplimab block programmed cell death 1 (PD-1), while atezolizumab, avelumab, and durvalumab inhibit PD-1 ligand (PD-L1) [2].

By blocking down-regulators of immunity, ICIs produce a widespread and persistent adaptive immune system activation. While enhancing the patient’s antitumor immune response, ICIs may trigger several immune-related adverse events (irAEs), which can involve any organ or system, including the nervous system [3, 4]. Neurologic immune-related adverse events (n-irAEs) are reported in 1–12% of patients treated with ICIs, and are associated with increased mortality and morbidity [5–7]. Given their rarity, details on epidemiology, clinical manifestations, and diagnostic findings of n-irAEs are mostly derived from literature reviews and meta-analyses of clinical trials [8–11], and there are few observational studies [6, 12, 13] evaluating patients in the real-life setting.

N-irAEs affecting the peripheral nervous system (PNS-irAEs), compared to those affecting the central nervous system (CNS-irAEs), are three times more common [8] and appear with a shorter latency [14]. ICI-induced neuromuscular events could manifest as neuropathies—involving both cranial and peripheral nerves—, neuromuscular junction disorders or myopathies [15]. The aim of this study was to investigate differences regarding latency of onset, associations with other immune-related toxicities, treatment response and outcomes among various PNS-irAEs phenotypes. With this in mind, we firstly describe epidemiological data, clinical manifestations, management, and outcomes of PNS-irAEs in a large real-life cohort of patients treated with ICIs at a single-center institution; subsequently, we compared patients with peripheral and/or cranial neuropathies and those with myasthenia gravis (MG) and/or myositis, with the aim of identifying distinctive features and outcomes.

## Materials and methods

### Patient selection and outcome measures

This was a single-center observational study, including a retrospective and a prospective cohort.

For the retrospective cohort of the study, we screened the medical data registries of patients treated with at least one ICI infusion at Sant’Orsola-Malpighi Hospital in Bologna between January 1, 2014, and December 31, 2020, and identified those who received at least one neurological evaluation. Additionally, we prospectively evaluated patients treated with ICIs between January 1, 2021, and March 31, 2022, and experienced new-onset neurological symptoms.

Finally, we identified patients who developed a n-irAE in the whole ICI-treated cohort (both retrospectively and prospectively). In accordance with the Consensus Disease Definition [16], we considered as n-irAEs the following conditions: (a) neurological symptoms beginning within 12 months of the *last* ICI infusion, after exclusion of other potential etiologies by a tailored diagnostic work-up, or (b) exacerbation of stable and well-controlled pre-existing neuro-immunologic conditions (e.g., MG). Among patients with n-irAEs, we selected those with peripheral nervous system involvement. In particular, MG was diagnosed in the presence of supportive examination findings (i.e., fluctuating ptosis, ocular, bulbar, axial or limb muscle weakness) plus anti-AchR antibodies positivity and/or neurophysiological evidence of impaired neuromuscular transmission (i.e., > 10% CMAP amplitude decrement at repetitive nerve stimulation, RNS). Myositis was diagnosed when patients with supportive clinical findings (i.e., fixed cranial, bulbar, ventilatory or limb muscle weakness) had EMG signs of irritable myopathy (i.e., fibrillation potential and/or positive sharp waves) and elevated CK. When criteria for both the diseases were respected, patients were diagnosed with concomitant MG/myositis. Patients who developed a n-irAE involving the central nervous system (CNS-irAEs) were not included in the analysis.

Clinical and ancillary data (electromyography, nerve conduction studies, autonomic function testing, laboratory exams) were extracted either retrospectively—by review of medical records—or collected prospectively. Motor and sensory nerve conduction studies were interpreted according to EFNS/PNS guidelines [17].

Follow-up time was considered from PNS-irAE development to death or to the end of the study period, for survived patients. Outcome measure was evaluated with the Modified Rankin Scale (mRS) [18], which was collected at the clinical nadir and after management of PNS-irAEs. For patients with MG, MGFA clinical classification score (at nadir and after treatment) was also collected. Patients with a reduction in mRS ≥ 1 were considered to have an improved outcome. On the contrary, patients were considered to have a fatal outcome if death was the result of the dysfunction of an organ affected by the immune-related toxicity.

PNS-irAEs phenotypes were classified a priori into two groups, according to the different anatomical site of involvement: (1) peripheral nerve (i.e., cranial/peripheral neuropathies) and (2) neuromuscular junction and /or skeletal muscle (i.e., myasthenia gravis [MG] and/or myositis).

The study was approved by the local Ethics Committee. Anonymized data will be shared upon request from any qualified investigator.

### Statistical analysis

Descriptive analysis is presented as mean (± standard deviation) for normally distributed data and median (and range) for data with non-normal distribution. The verification of normality in the distribution of variables is examined through the Shapiro–Wilk test.

Categorical data are presented as absolute and relative frequencies. Comparisons between groups were performed using *χ*^2^ test (or Fisher’s exact test, when appropriate) for categorical data and Mann–Whitney *U* test (or *t-*test, when appropriate) for continuous variables.

Comparison of pre- and post-management mRS values was performed with Wilcoxon’s non-parametric Signed rank test for paired data. Statistical analyses were performed using IBM SPSS Statistics Software (V.25.0). A *p* value < 0.05 was considered significant.

## Results

A total of 920 cancer patients (58% males, mean age 66.4 years [± 12.9]) received at least one ICI infusion during the study period (467 patients retrospectively collected from January 2014 to December 2020 and 453 patients prospectively enrolled from January 2021 to March 2022). Data concerning tumor types and ICI class used are presented in Table 1.

**Table 1:** Demographic characteristics of the entire treated population and PNS-irAEs patients

Characteristic	Patients with PNS-irAES (*n* = 20)	Patients without PNS-irAEs (*n* = 900)	Total (*n* = 920)
Sex, male	15 (75%)	524 (58%)	539 (59%)
Age, y mean (± SD)	70.8 (± 9.3)	60.23 (± 20.3)	66.4 (± 12.9)
Tumor type			
Melanoma	9 (45%)	264 (29.3%)	273 (29.6%)
NSCLC	5 (25%)	413 (45.9%)	418 (45.4%)
MCC	2 (10%)	4 (0.4%)	6 (0.7%)
Kidney	1 (5%)	68 (7.6%)	69 (7.5%)
SCLC	1 (5%)	33 (3.7%)	34 (3.7%)
Bladder	1 (5%)	18 (2%)	19 (2.1%)
Stomach	1 (5%)	4 (0.4%)	5 (0.5%)
Other types	0	192 (21.3%)	192 (21.3%)
ICI class used			
PD-1	13 (65%) Pembrolizumab (9) Nivolumab (4)	711 (78.8%)	724 (78.7%)
PD-L1	4 (20%) Avelumab (2) Atezolizumab (1) Durvalumab (1)	131 (14.7%)	135 (14.7%)
CTLA-4	2 (10%, Ipilimumab)	45 (5%)	47 (5.1%)
PD-1 + CTLA-4	1 (5%, Nivolumab + Ipilimumab)	13 (1.5%)	14 (1.5%)

*CTLA-4* cytotoxic T-lymphocyte antigen 4, *ICI* immune checkpoint inhibitor, *irAEs* immune-related adverse events, *MCC* Merkel cell carcinoma, *NSCLC* non-small cell lung cancer, *PD-1* programmed cell death 1, *PD-L1* programmed cell death ligand, *PNS* peripheral nervous system, *SCLC* small cell lung cancer

**Fig. 1:**
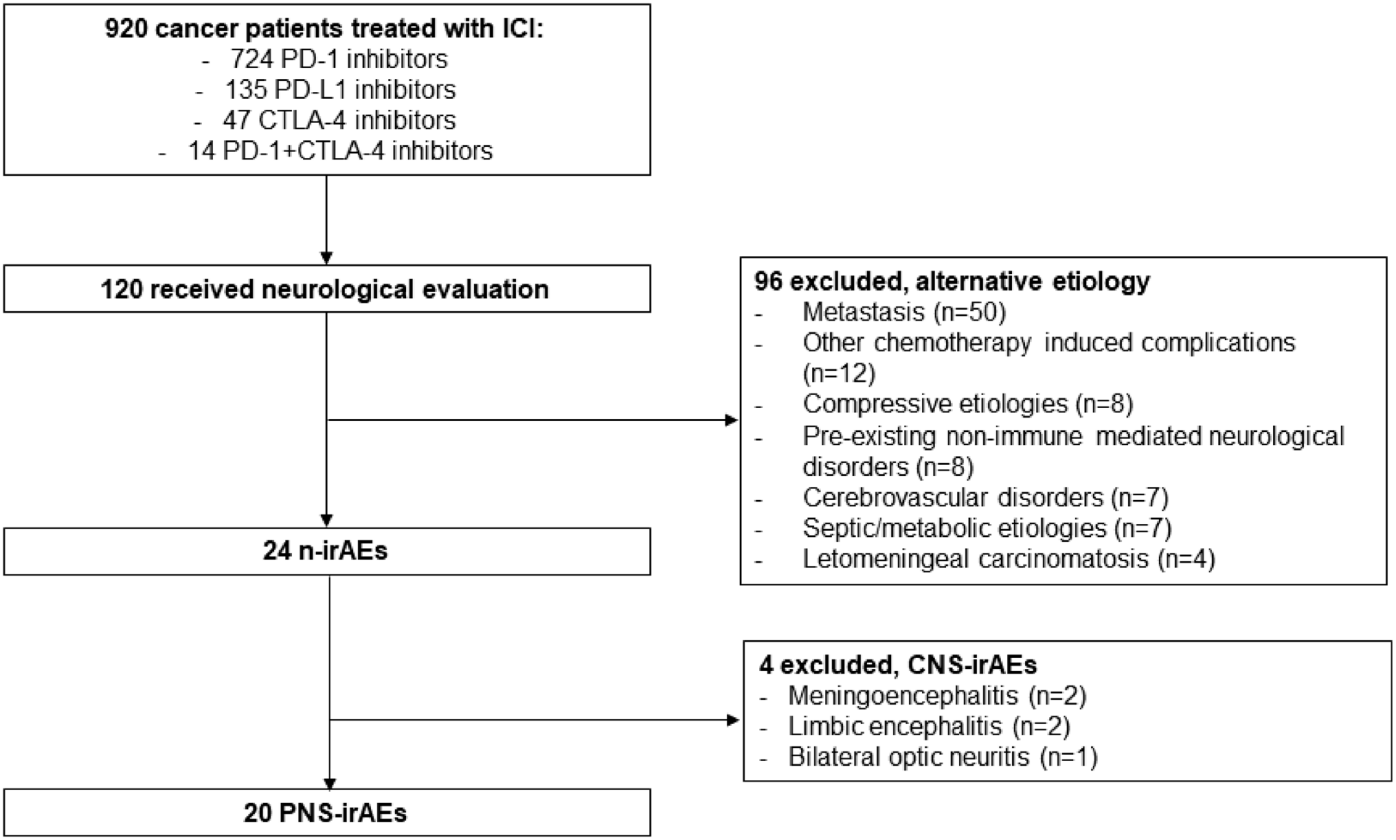
Patients’ selection. *CNS-irAEs* central nervous system immune-related adverse events, *CTLA-4* cytotoxic T-lymphocyte antigen 4, *ICI* immune checkpoint inhibitor, *n-irAEs* neurological immune-related adverse events, *PD-1* programmed cell death 1, *PD-L1* programmed cell death ligand, *PNS-irAEs* peripheral nervous system immune-related adverse events

Overall, 24 patients developed a n-irAEs. Four patients had a CNS-irAEs (two meningoencephalitis, one limbic encephalitis and one optic neuritis) and were not therefore included in the analysis. PNS-irAEs occurred in 20 patients (75% males, median age 72.9 years, range 55.9–83.3]), 11 in the retrospective cohort and 9 in the prospective one, accounting for an overall incidence of PNS-irAEs of 2.17% (Fig. 1).

The median time from ICI initiation to symptom onset was 8.8 weeks (range 2–59), while the median time from symptoms onset to clinical nadir was 3.5 weeks (range 1–6). Among patients with PNS-irAEs, underlying malignancies included melanoma (9 cases, 45%), NSCLC (5 cases, 25%), Merkel cell carcinoma (2 cases, 10%), bladder urothelial cancer (1 case, 5%), small cell lung cancer (SCLC, 1 case, 5%), ccRCC (1 case, 5%) and stomach cancer (1 case, 5%). Thirteen patients (65%) received PD-1 inhibitors, four (20%) PD-L1 inhibitors, two (10%) CTLA-4 inhibitors; one (5%) patient was treated with a combination of a PD-1 inhibitor and a CTLA-4 inhibitor. The frequency of PNS-irAEs in the four ICI groups listed above was 1.8%, 2.9%, 4.3%, and 7.1%, respectively. None of the patients had a known history of neurological or autoimmune disorders prior to ICI administration, except one patient with mild and well-controlled anti-acetylcholine receptor (AchR) antibody-positive generalized MG. Twelve (60%) patients developed concurrent non-neurologic irAEs, involving one (*n* = 6) or more than one (*n* = 6) organs (myocarditis, *n* = 4; thyroiditis, *n* = 4; hepatitis, *n* = 4; dermatitis, *n* = 3; adrenal insufficiency, *n* = 2; enterocolitis, *n* = 1; type 1 diabetes mellitus with anti-GAD antibodies, *n* = 1). The median follow-up time was 7 months (range 0–33).

### PNS-irAEs phenotypes

#### Neuropathies

Among the 20 patients with PNS-irAEs, 11 developed an immune-mediated cranial or peripheral neuropathy (Table 2). Seven (64%) were male, and the median age was 62.2 years (range 57.6–82.3). The median time from ICI initiation to n-irAEs onset was 19.1 weeks (range 1–59) and the median time to clinical nadir was 4 weeks (range 1–6).

**Table 2:** Characteristics of patients with ir-Neuropathies

Patient	Phenotype	Latency from ICI initiation (weeks)	Time to nadir (weeks)	Tumor	ICI	Chemotherapies	Other irAEs	mRS (nadir, post-management)	Management			Outcome (cause)	Follow-up (months)
									Immunomodulatory/suppressive treatment	ICI withdrawal	ICI rechallenge		
1	Polyneuropathy	5	4	SCLC	Atezolizumab	Concomitant (carboplatin, etoposide)	No	4 → 2	CCS	No	NA	Death (CP)	5
2	Polyneuropathy	3	3	MCC	Avelumab	No	No	3 → 1	CCS	No	NA	Death (CP)	3
3	Polyneuropathy	20	6	NSCLC	Pembrolizumab	No	No	3 → 0	CCS	Yes	Yes	Alive	6
4	Polyneuropathy	59	5	Melanoma	Nivolumab	No	No	4 → 3	CCS, IVIg	Yes	No	Death (CP)	28
5	Cranial neuropathy	8	1	Melanoma	Ipilimumab	No	Dermatitis	2 → 1	CCS	Yes	Yes	Death (CP)	25
6	Cranial neuropathy	22	1	Melanoma	Ipilimumab	Previous (dacarbazine)	Colitis	4 → 4	CCS	Yes	No	Death (CP)	4
7	Cranial neuropathy	28	2	Melanoma	Pembrolizumab	Previous (vemurafenib, cobimetinb)	No	2 → 2	CCS	Yes	No	Alive	10
8	Small fiber neuropathy	8	4	Melanoma	Nivolumab	No	No	2 → 1	No	Yes	No	Death (CP)	2
9	Small fiber neuropathy	17	6	Stomach	Pembrolizumab	Previous (docetaxel, oxaliplatin, 5-FU)	Thyroiditis	2 → 0	No	Yes	Yes	Alive	33
10	Small fiber neuropathy	19	4	NSCLC	Pembrolizumab	No	No	3 → 1	CCS	Yes	No	Alive	12
11	Brachial plexopathy	20	1	Bladder	Pembrolizumab	Previous (platinum-derived CT)	Dermatitis	3 → 0	CCS	Yes	Yes	Alive	12

*CCS* corticosteroids, *CP* cancer progression, *IDP* inflammatory demyelinating polyneuropathy, *ICI* immune checkpoint inhibitor, *irAEs* immune-related adverse events, *IVIg* intravenous immunoglobulins, *F* female, *M* male, *MCC* Merkel cell carcinoma, *mRS* modified Rankin scale, *NSCLC* non-small cell lung carcinoma, *SCLC* small cell lung cancer, *NA* not applicable

Neuropathy phenotypes were polyneuropathy (*n* = 4), cranial neuropathy (*n* = 3), small-fiber neuropathy (*n* = 3) and brachial plexopathy (*n* = 1).

Four patients developed a sensory-motor polyneuropathy demonstrated at clinical examination by symmetric proximal and distal muscle weakness predominant in the lower limbs, distal sensory loss and decreased or absent deep tendon reflexes. Symptoms developed insidiously and led to a moderate disability over 2–3 weeks (i.e., inability to lift from the chair autonomously and to walk unassisted). Cerebrospinal fluid (CSF) examination revealed albumin-cytologic dissociation in all patients (mean white blood cells = 7/mm^3^ (± 2.3), n.v. < 5/mm^3^; mean protein levels = 74 mg/dL (± 16.7), n.v. < 50 mg/dL). Nerve conduction studies were strongly supportive of demyelination (i.e., increased distal motor latencies, reduced motor and sensory conduction velocities, increased F-waves latencies) in all four patients. Antiganglioside antibodies were searched in three patients and resulted negative.

Three patients developed cranial neuropathies: two presented with unilateral peripheral facial palsy, in one case associated with bilateral vestibulocochlear neuropathy, in the other with burning mouth syndrome; one patient developed bilateral vestibulocochlear neuropathy.

Three patients developed small-fiber neuropathy (SFN). Patients presented with subacute onset of painful paresthesia, anhidrosis, and reduced pain and heat sensation. Two patients were previously treated with potentially neurotoxic platinum-derived chemotherapy prior to ICI. Nevertheless, symptoms developed a long period after the completion of chemotherapy (eight months in one patient and two years in the other), thus making an etiological relationship unlikely. Electrodiagnostic studies showed absence of the skin sympathetic response and normal nerve conductions. Skin biopsy was not performed.

One patient developed right brachial plexopathy, manifesting with acute onset painless weakness of the right arm. Electrodiagnostic studies were consistent with a right brachial pan-plexopathy. CSF analysis revealed pleocytosis (white blood cells: 16/mm^3^) and increased protein levels (159 mg/dL).

Four (36%) patients developed a concurrent non-neurologic ir-AE (dermatitis, *n* = 2; thyroiditis, *n* = 1; enterocolitis, *n* = 1).

Overall, patients with ir-neuropathies had a median mRS score of 3 at disease peak (range 2–4).

In nine patients (82%), ICI treatment was discontinued after n-irAEs presentation. Among them, seven were treated with oral prednisone (0.5–1 mg/kg/die), and one was subsequently escalated to intravenous immunoglobulin (IVIg, 0.4 g/kg/day for five days, followed by a monthly maintenance infusion). Two patients, both with SFN, did not receive immunosuppressive therapy, as symptoms improved after ICI withdrawal and symptomatic treatment.

In four patients (one with polyneuropathy, one with cranial neuropathy, one with SFN and one with brachial plexopathy), a rechallenge of ICI therapy was attempted after achieving a complete remission of neurological symptoms. Median duration from symptoms onset to retreatment was 8 months (range 2.5–12). No patient exhibited neurological relapses after ICI rechallenge.

In the two remaining patients, who had mild/moderate demyelinating polyneuropathy and no further cancer treatment options, ICI was continued—along with low-dose prednisone—under a strict neurological follow-up, without worsening of neurological symptoms.

Neurological symptoms improved in nine patients (82%) and remained stable in two (one facial palsy and one with bilateral vestibulocochlear neuropathy). Follow-up nerve conduction studies were performed in three patients with demyelinating polyneuropathy and showed improvement of motor and sensory conduction velocities. None of the patients died due to ir-neuropathy. Overall, immunosuppressive treatment and/or ICI withdrawal were associated with a significant mRS improvement (median mRS at nadir = 3 [range 2–4], median mRS post-management = 1 [range = 0–2], *p* = 0.004).

#### Myasthenia gravis and/or myositis

Nine patients developed MG and/or myositis (Table 3). Eight (89%) were men and the median age was 73.7 years (range 67–80). The median latency from ICI initiation and n-irAEs development was 6.6 weeks (range 1–29) and the median time to clinical nadir was 3 weeks (range 1–4).

**Table 3:** Characteristics of patients with ir-MG/myositis

Patient	Phenotype	Latency from ICI initiation (weeks)	Time to nadir (weeks)	Tumor	ICI	Chemotherapies	Other irAEs	mRS (nadir, post-management)	MGFA (nadir, post-management)	Management		Outcome	Follow-up (months)
										Immuno-modulatory/suppressive treatment	ICI withdrawal		
1	MG/myositis	2	1	Melanoma	Pembrolizumab	No	Myocarditis, transaminitis	5 → 6	IVB → death	CCS	Yes	Death (irAE)	0.5
2	MG/myositis	3	2	Merkel	Avelumab	Previous (carboplatinum, etoposide)	Thyroiditis	3 → 0	IIIA → I	CCS	Yes	Death (CP)	12
3	MG/myositis	5	1	Melanoma	Pembrolizumab	No	Myocarditis	3 → 0	IIIA → IIA	CCS	Yes	Alive	5
4	MG/myositis	7	2	NSCLC	Durvalumab	Previous (platinum-derived CT)	Myocarditis, Transaminitis	5 → 6	V → death	CCS, IVIg	Yes	Death (irAE)	17
5	MG/myositis	8	4	Melanoma	Nivolumab + Ipilimumab	No	Myocarditis, Transaminitis, DM type 1 (with anti-GAD antibodies)	5 → 6	V → death	CCS, IVIg	Yes	Death (irAE)	1
6	MG/myositis	19	4	NSCLC	Pembrolizumab	Concomitant (carboplatin and paclitaxel)	Thyroiditis, dermatitis	2 → 0	IIA	CCS	Yes	Alive	7
7	Myositis	3	2	Melanoma	Nivolumab	No	Hepatitis, Thyroiditis	3 → 0	NA	CCS	Yes	Alive	16
8	Myositis	29	3	NSCLC	Pembrolizumab	No	Adrenal insufficiency	3 → 1	NA	CCS	Yes	Alive	7
9	MG^1^	7	4	Kidney	Nivolumab	Previous (sunitinib; cabozatinib)	No	5 → 6	V → death	PLEX	Yes	Death (irAE)	1

*CCS* corticosteroids, *CP* cancer progression, *DM* diabetes mellitus, *GAD* glutamic acid decarboxylase, *ICI* immune checkpoint inhibitor, *irAE* immune-related adverse event, *IVIg* intravenous immunoglobulins, *MG* myasthenia gravis, *NA* not applicable, *NSCLC* non-small cell lung carcinoma, *PLEX* plasmapheresis

^1^MG exacerbation

Six patients (67%) developed concomitant MG and myositis. Patients presented with fatigable signs of bulbar involvement (dysphagia, dysarthria, and dyspnea), ophthalmoplegia, and limb-girdle pattern muscle weakness with myalgias; in three patients, the first complaint was “dropped head” due to neck extensors weakness.

Two patients had isolated myositis, manifesting with proximal muscle weakness and myalgia. One patient had an abrupt exacerbation of a pre-existing and previously well-controlled MG after two infusions of nivolumab.

Eight patients (89%) developed a concurrent non-neurologic ir-AE (myocarditis, *n* = 4; thyroiditis, *n* = 2; hepatitis, *n* = 2; adrenal insufficiency, *n* = 1; diabetes mellitus, *n* = 1).

Creatin kinase (CK) levels were elevated in patients with myositis (± MG), ranging from 380 to 21,700 IU/L (mean value: 6,551 UI/L). All patients with MG tested positive for anti-AchR antibodies (mean titer: 1.84 nmol/L), while three patients with MG/myositis and concurrent myocarditis had anti-titin antibodies. Electrodiagnostic studies were performed in seven patients (five with MG/myositis, two with isolated myositis). On EMG examination, patients with myositis presented abnormal spontaneous activity (positive sharp waves and fibrillation potentials) and short-duration and low-amplitude motor unit action potentials. Repetitive nerve stimulation was consistent with post-synaptic dysfunction in two patients with MG/myositis. Muscle biopsy was performed in one patient and showed inflammatory changes without prominent necrosis.

Overall, patients with MG/myositis had a median mRS score of 3 at disease peak (range 3–5). MGFA clinical classification categories at disease peak ranged from MGFA-IIB to MGFA-V.

Eight patients (89%) were treated with steroids: four with oral prednisone (1 mg/kg/day), three with intravenous methylprednisolone (0.75 mg/kg/day), one with high-dose intravenous methylprednisolone pulse (1 g/day for 3 days). Two patients were subsequently escalated to intravenous immunoglobulin (IVIg) after steroids. One patient with myasthenic crisis received plasmapheresis (PLEX, 5 cycles). Treatment with ICIs was discontinued in all patients, and none was rechallenged.

The median follow-up was 6 months (range 0–17). Immunosuppressive therapy and ICI withdrawal determined an improvement of neurological symptoms in five (56%) patients. Immune-related toxicities led to death four (44%) patients, three with MG/myositis/myocarditis and one with MG. Two patients died because of neuromuscular ventilatory dysfunction; two patients, both with MG/myositis/myocarditis, died from sudden cardiac arrest due to malignant arrhythmia.

Overall, immunosuppression and ICI withdrawal were not associated with significant reduction of mRS (median mRS at nadir = 3 [range 3–5], median mRS post-management 1 [range 0–6], *p* = 0.11).

**Fig. 2:**
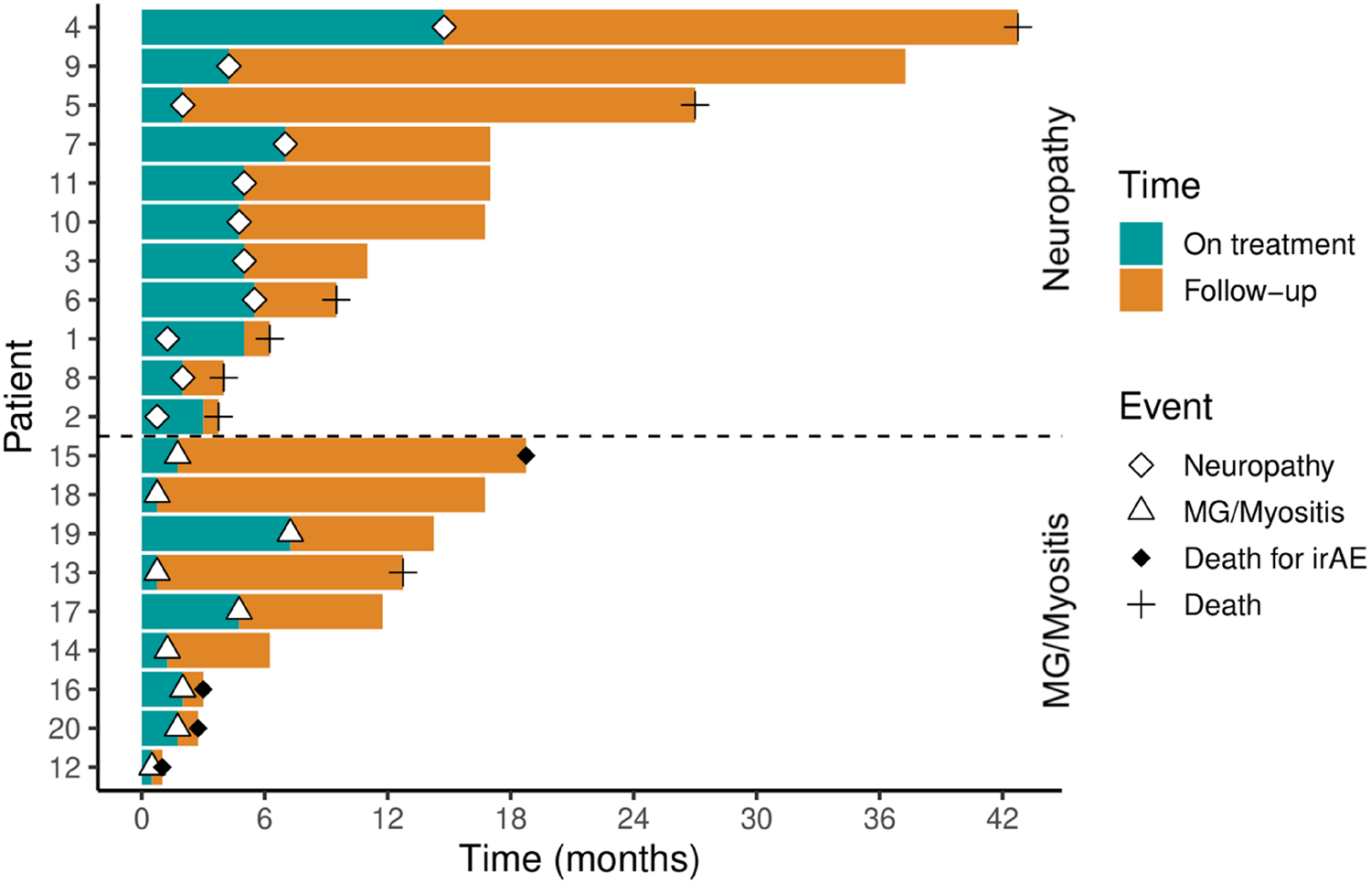
Swimmer plot graph that shows the duration of ICI treatment (blue part of the bar) and total follow-up time (orange part of the bar) in patients with ir-neuropathies (above the dotted line) and ir-MG/myositis (under the dotted line). The onset of the PNS-irAEs is marked. *MG* myasthenia gravis

Time of onset, outcome, and follow-up time of the patients with ir-neuropathies and ir-MG/myositis are summarized in Fig. 2.

### Comparison between patients with neuropathies and MG/myositis

A comparison between patients with neuropathies and MG/myositis is shown in Table 4. MG/myositis patients were numerically more commonly men (89% vs*.* 64%, *p* = 0.32) and older (median = 73 vs. 62, *p* = 0.34) than those with neuropathies. There were no differences in tumor type or ICI class used.

**Table 4:** Comparison between patients with neuropathies and patients with MG/myositis

Characteristic	MG/myositis *N* = 9 (45%)	Neuropathies *N* = 11 (55%)	*p* value
Male sex, *n*	8 (88.9%)	7 (63.6%)	0.319
Age, years median [range]	73.7 [67–80]	62.2 [57.6–82.3]	0.342
Other irAEs, *n*	8 (88.9%)	4 (36.4%)	**0.025**
Tumor, *n* (%)			
Melanoma	4 (44.5%)	5 (45.4%)	0.999
NSCLC	3 (33.3%)	2 (18.2%)	
Kidney	1 (11.1%)	0 (0%)	
Bladder	0 (0%)	1 (9.1%)	
MCC	1 (11.1%)	1 (9.1%)	
SCLC	0 (0%)	1 (9.1%)	
Stomach	0 (0%)	1 (9.1%)	
ICI class, *n*			
PD-1	6 (66.7%)	7 (63.6%)	0.622
PD-L1	2 (22.2%)	2 (18.2%)	
CTLA-4	0 (0%)	2 (18.2%)	
PD-1 + CTLA-4	1 (11.1%)	0 (0%)	
Time to onset, weeks [range]	6.6 [1–29]	19.1 [1–59]	**0.036**
Time to clinical nadir, weeks [range]	4 [1–6]	3 [1–4]	0.584
mRS nadir [range]	3 [3–5]	3 [2–4]	0.104
mRS post-management [range]	1 [0–6]	1 [0–2]	0.693
Outcome, *n*			
Death	4 (44.4%)	0 (0%)	**0.026**
Stable/improved	5 (55.6%)	11 (100%)	

*CTLA-4* cytotoxic T-lymphocyte antigen 4, *ICI* immune checkpoint inhibitor, *irAEs* immune-related adverse events, *MCC* Merkel cell carcinoma, *MG* myasthenia gravis, *mRS* modified Rankin scale, *NSCLC* non-small cell lung cancer, *PD-1* programmed cell death 1, *PD-L1* programmed cell death ligand, *PNS* peripheral nervous system, *SCLC* small cell lung cancer

Values in bold are statistically significant

Concurrent non-neurologic ir-AEs were significantly more common in patients with MG/myositis than in those with neuropathies (89% vs 36%, respectively; *p* = 0.028). In addition, time from ICI initiation to onset of PNS-irAEs was significantly shorter in patients with MG/myositis than in those with neuropathies (6.6 [3.0–7.6] vs 19.1 [8.0–21.7] weeks, respectively; *p* = 0.036). The median time from symptoms onset to clinical nadir was similar between the two groups (Neuropathies = 4 [1–6] vs*.* MG/Myositis = 3 [1–4] weeks, *p* = 0.584). Lastly, more patients with MG/myositis died of irAEs-related causes than those with neuropathies (44% vs 0%, respectively; *p* = 0.026).

## Discussion

In the present study, we described clinical, diagnostic, and outcome characteristics of PNS-irAEs in a large cohort of cancer patients treated with ICIs between January 2014 and March 2022 at our Institution. The frequency of PNS-irAEs in the overall cohort resulted to be 2.17%. A systematic review of published clinical trials reported the frequency of n-irAEs to be below 1% [5]. Available retrospective studies on large cohorts of ICI-treated patients identify a frequency of 1.5 and 1.22% for all n-irAEs (including both PNS and CNS-irAEs) [6, 19] and of 0.6% for severe neuromuscular adverse events [20]. Indeed, our data suggest that the frequency of ICI-induced neuromuscular adverse events may be higher than previously reported.

We observed that in our cohort MG/myositis patients, compared to those with peripheral/cranial neuropathies present (1) a shorter time to onset from ICI initiation, (2) a more common association with concurrent non-neurologic irAEs and (3) a more aggressive clinical course, exhibiting a less satisfactory response to immunosuppressive treatments with a higher mortality rate.

Immunopathogenic mechanisms underlying n-irAEs pathophysiology are still to be fully elucidated. By altering the equilibrium between T-regulator and T-effector cells, ICI has been shown to induce a loss of peripheral tolerance, which may promote epitope sharing between cancer cells and neurons and, eventually, a cross-reaction against self-tissues [21]. Additionally, a subset of predisposed patients may have a pre-existing subset of T-cells autoreactive to self-antigens latent in peripheral blood, whose potential pathogenic role is effectively controlled by the pathways of immune checkpoints. Inhibition of these mechanisms by ICIs may trigger the activation of both CD8^+^—thus causing cytotoxic attack against skeletal muscle and CD4^+^ T cells [22], leading in turn to the activation of B-cells and, eventually, to the production of autoreactive antibodies (i.e., anti-AchR and anti-skeletal muscle antibodies). This hypothesis is supported by patients with ir-MG in which the detection of anti-AchR antibodies in serum samples taken prior to ICI initiation, yielded positive results [23, 24]. Our patient with mild and well-controlled anti-AchR positive MG who abruptly deteriorated after two infusions of nivolumab, leading to a fatal myasthenic crisis, is in keep with this hypothesis. These findings suggest that patients with pre-existing anti-AchR antibodies are at higher risk of developing ir-MG (or exacerbating it, if pre-existing). On the other hand, the report of an anti-AchR seropositive patient who had favourable cancer outcome without developing MG after anti-PD1 therapy, suggest that ICI could be safely administered in asymptomatic anti-AchR seropositive cancer patients [25].

Bearing all this in mind, we believe that anti-AchR antibody testing might be considered as a routinary screening measure in cancer patients prior to ICI therapy. An eventual anti-AchR positivity, however, should not be deemed as an absolute contraindication to ICI therapy, but should prompt a close clinical monitoring, consisting in serial neurological evaluation (e.g., before every single ICI cycle) and CK dosage, with the aim of identifying precocious signs of neuromuscular dysfunction.

Patients with MG/myositis more frequently developed concurrent non-neurologic irAEs, indicating the activation of a more widespread autoimmune process in this group. Notably, four out of six patients with MG/myositis presented with concurrent myocarditis. The association of immune-related MG, myositis, and myocarditis has been identified as a specific overlap syndrome (IM3OS) [26], characterized by a short latency of onset from ICI initiation—on average after one dose—and an abrupt and clinical deterioration, often requiring admission to intensive care unit [27]. The co-occurrence of myocarditis [28] and the elevation of serum troponin [29] have been identified as predictors of poor prognosis in patients with ir-MG/myositis and IM3OS is characterized by a high mortality (up to 60% of cases) [30], which can occur due to both cardiac (i.e., arrythmia) and neuromuscular (i.e., respiratory failure) involvement. On the other hand, concomitant myositis or myocarditis is rarely observed in idiopathic MG and is usually reported in cases of thymoma-associated MG [31, 32]. Interestingly, we detected anti-titin antibodies in three out of four patients with MG/myositis/myocarditis (75%), confirming previous reports [33, 34]. Anti-titin antibodies have been shown to bind in vitro to skeletal and heart muscle tissue [35], but a pathogenic role in vivo has never been demonstrated. Anti-titin antibodies are detected in 68–95% of patients with thymoma-associated MG [36, 37] and are common in late-onset (> 60 years) non-thymoma MG patients [38, 39]. Moreover, they are found in thymoma patients without MG [40], suggesting that the occurrence of thymoma *in itself* could alter titin presentation to T cells. Taken as a whole, clinical (i.e., association with myositis and myocarditis) and laboratory (i.e., frequent anti-titin positivity) similarities between ICI-induced and thymoma-associated autoimmunity, may led us to speculate that both this conditions (thymoma and ICI treatment) produce a common immunological dysregulation, which may deal with altered autoantigens presentation and antigen spreading at the neuromuscular junction [23].

Most patients in our series received immunosuppressive/immunomodulatory treatments and had ICI withdrawn, in accordance with current guidelines [41].

Patients with neuropathies showed an overall favourable response, except for two patients with vestibulocochlear neuropathy (one with associated facial palsy), confirming the already observed steroid-refractoriness of ICI-induced vestibulocochlear neuropathy [42].

Conversely, patients with MG/myositis did not show an overall favourable response and four died despite the institution of prompt immunosuppressive treatment. Our series therefore confirms that patients who develop MG/myositis have a dismal prognosis, especially when associated with myocarditis. Current consensus guidelines provide different therapeutic approaches for MG and myositis [41]. However, considering that these conditions often present concomitantly, tailored approaches for overlap syndromes should be devised. Future studies, specifically addressing ir-MG/myositis overlapping conditions, are therefore urgently needed.

Finally, four patients with neuropathy were rechallenged with the same ICI after the resolution of neurological symptoms, and none exhibited neurological relapses. A recent review identified two relapses in 17 rechallenged patients with ir-neuropathy [10]. Therefore, also considering our findings, ICI-rechallenge could be evaluated in patients with ir-neuropathy, as relapse risk seems acceptable if compared with the potential antitumor efficacy of ICIs.

However, future larger prospective studies are needed to confirm this preliminary observation. Until then, the decision of ICI retreatment must be evaluated on a case-by-case basis by an experienced multidisciplinary team, considering previous oncological response to ICI, therapeutic alternatives, severity of neurological involvement at disease peak, and neurological sequelae.

The present study has some limitations. First, due to its partial retrospective nature, some patients with PNS-irAEs might have been overlooked (e.g., if symptoms were mild, thus not prompting neurological consultancy, or if the neurological evaluation was not performed at our institution). Second, the clinical impairment due to the PNS-irAE was evaluated using mRS, which is not specifically validated for addressing neuromuscular-related disability. Lastly, despite an overall large sample size of ICI-treated patients, the number of PNS-irAEs was limited, thus limiting the statistical power of the analysis between groups.

In conclusion, PNS-irAEs represent rare, potentially underestimated, complications of ICI treatment, which may present with a wide clinical spectrum. MG and myositis are often concomitant and, compared to neuropathies, tend to appear with a shorter latency from ICI exposure and are more frequently associated with non-neurologic irAEs. While patients with neuropathies typically show a favorable neurological outcome, those with MG/myositis may show an aggressive clinical course and a potential fatal outcome, in particular when associated with myocarditis.

Additional research is needed to further characterize n-irAEs pathophysiology, to identify tailored therapeutic approaches for overlapping immune-related toxicities and to detect biomarkers capable of identifying patients at risk of developing severe n-irAEs prior to ICI initiation.

## Data Availability

The corresponding author has full access to all of the data and has the right to publish any and all data separate and apart from any sponsor.

## References

[1] Robert C (2020). A decade of immune-checkpoint inhibitors in cancer therapy. Nat Commun.

[2] Bagchi S, Yuan R, Engleman EG (2021). Immune checkpoint inhibitors for the treatment of cancer: clinical impact and mechanisms of response and resistance. Annu Rev Pathol.

[3] Postow MA, Sidlow R, Hellmann MD (2018). Immune-related adverse events associated with immune checkpoint blockade. N Engl J Med.

[4] Pensato U, Guarino M, Muccioli L (2022). The role of neurologists in the era of cancer immunotherapy: focus on CAR T-cell therapy and immune checkpoint inhibitors. Front Neurol.

[5] Cuzzubbo S, Javeri F, Tissier M, Roumi A, Barlog C, Doridam J, Lebbe C, Belin C, Ursu R, Carpentier AF (2017). Neurological adverse events associated with immune checkpoint inhibitors: review of the literature. Eur J Cancer.

[6] Dubey D, David WS, Reynolds KL, Chute DF, Clement NF, Cohen JV, Lawrence DP, Mooradian MJ, Sullivan RJ, Guidon AC (2020). Severe neurological toxicity of immune checkpoint inhibitors: growing spectrum. Ann Neurol.

[7] Wang DY, Salem JE, Cohen JV, Chandra S, Menzer C, Ye F, Zhao S, Das S, Beckermann KE, Ha L, Rathmell WK, Ancell KK, Balko JM, Bowman C, Davis EJ, Chism DD, Horn L, Long GV, Carlino MS (2018). Fatal toxic effects associated with immune checkpoint inhibitors: a systematic review and meta-analysis. JAMA Oncol.

[8] Marini A, Bernardini A, Gigli GL, Valente M, Muñiz-Castrillo S, Honnorat J, Vogrig A (2021). Neurologic adverse events of immune checkpoint inhibitors: a systematic review. Neurology.

[9] Cuzzubbo S, Javeri F, Tissier M, Roumi A, Barlog C, Doridam J, Lebbe C, Belin C, Ursu R, Carpentier AF (2017). Neurological adverse events associated with immune checkpoint inhibitors: review of the literature. Eur J Cancer.

[10] Villagrán-García M, Velasco R (2022). Neurotoxicity and safety of the rechallenge of immune checkpoint inhibitors: a growing issue in neuro-oncology practice. Neurol Sci.

[11] Farooq MZ, Aqeel SB, Lingamaneni P, Pichardo RC, Jawed A, Khalid S, Banskota SU, Fu P, Mangla A (2022). Association of immune checkpoint inhibitors with neurologic adverse events: a systematic review and meta-analysis. JAMA Netw Open.

[12] Dubey D, David WS, Amato AA, Reynolds KL, Clement NF, Chute DF, Cohen JV, Lawrence DP, Mooradian MJ, Sullivan RJ, Guidon AC (2019). Varied phenotypes and management of immune checkpoint inhibitor-associated neuropathies. Neurology.

[13] Mancone S, Lycan T, Ahmed T, Topaloglu U, Dothard A, Petty WJ, Strowd RE (2018). Severe neurologic complications of immune checkpoint inhibitors: a single-center review. J Neurol.

[14] Bruna J, Argyriou AA, Anastopoulou GG, Alemany M, Nadal E, Kalofonou F, Piulats JM, Simó M, Velasco R, Kalofonos HP (2020). Incidence and characteristics of neurotoxicity in immune checkpoint inhibitors with focus on neuromuscular events: experience beyond the clinical trials. J Peripher Nerv Syst.

[15] Psimaras D, Velasco R, Birzu C, Tamburin S, Lustberg M, Bruna J, Argyriou AA (2019). Immune checkpoint inhibitors-induced neuromuscular toxicity: from pathogenesis to treatment. J Peripher Nerv Syst.

[16] Guidon AC, Burton LB, Chwalisz BK, Hillis JM, Schaller T, Reynolds KL (2021). Consensus disease definitions for the spectrum of neurologic immune related adverse events. J Clin Oncol.

[17] Van den Bergh PYK, van Doorn PA, Hadden RDM, Avau B, Vankrunkelsven P, Allen JA, Attarian S, Blomkwist-Markens PH, Cornblath DR, Eftimov F, Goedee HS, Harbo T, Kuwabara S, Lewis RA, Lunn MP, Nobile-Orazio E, Querol L, Rajabally YA, Sommer C, Topaloglu HA (2021). European Academy of Neurology/Peripheral Nerve Society guideline on diagnosis and treatment of chronic inflammatory demyelinating polyradiculoneuropathy: Report of a joint Task Force—Second revision. Eur J Neurol.

[18] Quinn TJ, Dawson J, Walters MR, Lees KR (2009). Reliability of the modified rankin scale: a systematic review. Stroke.

[19] Plaçais L, Michot J-M, Champiat S, Romano-Martin P, Baldini C, Joao MS, Marabelle A, Voisin A-L, Not A, Labeyrie C, Beaudonnet G, Laparra A, Maria ATJ, Masseau A, Dehette S, Deleporte A, Echaniz-Laguna A, Denier C, Adams D (2021). Neurological complications induced by immune checkpoint inhibitors: a comprehensive descriptive case-series unravelling high risk of long-term sequelae. Brain Commun.

[20] Hyun JW, Kim KH, Kim SH, Kim HJ (2022). Severe neuromuscular immune-related adverse events of immune checkpoint inhibitors at national cancer center in Korea. J Cancer Res Clin Oncol.

[21] Solimando AG, Crudele L, Leone P, Argentiero A, Guarascio M, Silvestris N, Vacca A, Racanelli V (2020). Immune checkpoint inhibitor-related myositis: from biology to bedside. Int J Mol Sci.

[22] Seki M, Kitano S, Suzuki S (2022). Neurological disorders associated with immune checkpoint inhibitors: an association with autoantibodies. Cancer Immunol Immunother.

[23] Suzuki S, Ishikawa N, Konoeda F, Seki N, Fukushima S, Takahashi K, Uhara H, Hasegawa Y, Inomata S, Otani Y, Yokota K, Hirose T, Tanaka R, Suzuki N, Matsui M (2017). Nivolumab-related myasthenia gravis with myositis and myocarditis in Japan. Neurology.

[24] Bilen MA, Subudhi SK, Gao J, Tannir NM, Tu SM, Sharma P (2016). Acute rhabdomyolysis with severe polymyositis following ipilimumab-nivolumab treatment in a cancer patient with elevated anti-striated muscle antibody. J Immunother Cancer.

[25] Saruwatari K, Sato R, Nakane S, Sakata S, Takamatsu K, Jodai T, Mito R, Horio Y, Saeki S, Tomita Y, Sakagami T (2019). The risks and benefits of immune checkpoint blockade in anti-AChR antibody-seropositive non-small cell lung cancer patients. Cancers.

[26] Pathak R, Katel A, Massarelli E, Villaflor VM, Sun V, Salgia R (2021). Immune checkpoint inhibitor-induced myocarditis with myositis/myasthenia gravis overlap syndrome: a systematic review of cases. Oncologist.

[27] Deharo F, Carvelli J, Cautela J, Garcia M, Sarles C, Maues de Paula A, Bourenne J, Gainnier M, Bichon A (2022). Immune checkpoint inhibitor-induced myositis/myocarditis with myasthenia gravis-like misleading presentation: a case series in intensive care unit. J Clin Med.

[28] Shi J, Tan Y, Huang Y, Li K, Yan J, Guan Y, Zhang L (2022). Association between clinical factors and result of immune checkpoint inhibitor related myasthenia gravis: a single center experience and systematic review. Front Neurol.

[29] Longinow J, Zmaili M, Skoza W, Kondoleon N, Marquardt R, Calabrese C, Funchain P, Moudgil R (2022). Immune checkpoint inhibitor induced myocarditis, myasthenia gravis, and myositis: a single-center case series. Cancer Med.

[30] Naqash AR, Moey MYY, Cherie Tan X-W, Laharwal M, Hill V, Moka N, Finnigan S, Murray J, Johnson DB, Moslehi JJ, Sharon E (2022). Major adverse cardiac events with immune checkpoint inhibitors: a pooled analysis of trials sponsored by the national cancer institute—cancer therapy evaluation program. J Clin Oncol.

[31] Paik JJ, Corse AM, Mammen AL (2014). The co-existence of myasthenia gravis in patients with myositis: a case series. Semin Arthritis Rheum.

[32] Uchio N, Taira K, Ikenaga C, Kadoya M, Unuma A, Yoshida K, Nakatani-Enomoto S, Hatanaka Y, Sakurai Y, Shiio Y, Kaida K, Kubota A, Toda T, Shimizu J (2019). Inflammatory myopathy with myasthenia gravis: thymoma association and polymyositis pathology. Neurol Neuroimmunol NeuroInflamm.

[33] Haddox CL, Shenoy N, Shah KK, Kao JC, Jain S, Halfdanarson TR, Wijdicks EF, Goetz MP (2017). Pembrolizumab induced bulbar myopathy and respiratory failure with necrotizing myositis of the diaphragm. Ann Oncol.

[34] Takamatsu K, Nakane S, Suzuki S, Kosaka T, Fukushima S, Kimura T, Miyashita A, Mukaino A, Yamakawa S, Watanabe K, Jinnin M, Komohara Y, Ihn H, Ando Y (2018). Immune checkpoint inhibitors in the onset of myasthenia gravis with hyperCKemia. Ann Clin Transl Neurol.

[35] Suzuki S, Utsugisawa K, Nagane Y, Suzuki N (2011). Three types of striational antibodies in myasthenia gravis. Autoimmune Dis.

[36] Aarli JA, Stefansson K, Marton LSG, Wollmann RL (1990). Patients with myasthenia gravis and thymoma have in their sera IgG autoantibodies against titin. Clin Exp Immunol.

[37] Romi F, Skeie GO, Gilhus NE, Aarli JA (2005). Striational antibodies in myasthenia gravis. Arch Neurol.

[38] Yamamoto AM, Gajdos P, Eymard B, Tranchant C, Warter J-M, Gomez L, Bourquin C, Bach J-F, Garchon H-J (2001). Anti-titin antibodies in myasthenia gravis. Arch Neurol.

[39] Somnier FE, Engel PJH (2002). The occurrence of anti-titin antibodies and thymomas: a population survey of MG 1970–1999. Neurology.

[40] Vernino S, Lennon VA (2004). Autoantibody profiles and neurological correlations of thymoma. Clin Cancer Res.

[41] Schneider BJ, Naidoo J, Santomasso BD, Lacchetti C, Adkins S, Anadkat M, Atkins MB, Brassil KJ, Caterino JM, Chau I, Davies MJ, Ernstoff MS, Fecher L, Ghosh M, Jaiyesimi I, Mammen JS, Naing A, Nastoupil LJ, Phillips T (2021). Management of immune-related adverse events in patients treated with immune checkpoint inhibitor therapy: ASCO guideline update. J Clin Oncol.

[42] Vogrig A, Muñiz-Castrillo S, Joubert B, Picard G, Rogemond V, Skowron F, Egri M, Desestret V, Tilikete C, Psimaras D, Ducray F, Honnorat J (2021). Cranial nerve disorders associated with immune checkpoint inhibitors. Neurology.

